# Tongue-on-a-Chip: Parallel Recording of Sweet and Bitter Receptor Responses to Sequential Injections of Pure and Mixed Sweeteners

**DOI:** 10.1021/acs.jafc.4c00815

**Published:** 2024-07-01

**Authors:** Margriet Roelse, Nadejda Krasteva, Steve Pawlizak, Michaela K. Mai, Maarten A. Jongsma

**Affiliations:** † BU Bioscience, Wageningen University and Research, Droevendaalsesteeg 1, 6708 PB Wageningen, The Netherlands; ‡ Stuttgart Laboratory 2, Sony Semiconductor Solutions Europe, Sony Europe B.V., Zweigniederlassung Deutschland, Hedelfinger Str. 61, 70327 Stuttgart, Germany

**Keywords:** microfluidics, GPCR, taste threshold, EC50, lingering, onset, off-taste, bitter blocking, antagonism

## Abstract

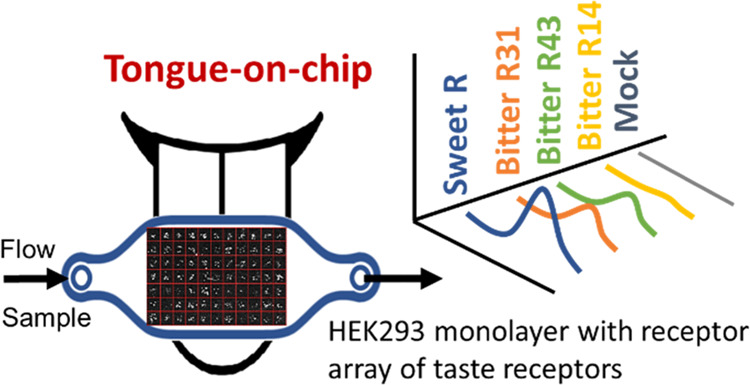

A microfluidic tongue-on-a-chip platform has been evaluated relative to the known sensory properties of various sweeteners. Analogous metrics of typical sensory features reported by human panels such as sweet taste thresholds, onset, and lingering, as well as bitter off-flavor and blocking interactions were deduced from the taste receptor activation curves and then compared. To this end, a flow cell containing a receptor cell array bearing the sweet and six bitter taste receptors was transiently exposed to pure and mixed sweetener samples. The sample concentration gradient across time was separately characterized by the injection of fluorescein dye. Subsequently, cellular calcium responses to different doses of advantame, aspartame, saccharine, and sucrose were overlaid with the concentration gradient. Parameters describing the response kinetics compared to the gradient were quantified. Advantame at 15 μM recorded a significantly faster sweetness onset of 5 ± 2 s and a longer lingering time of 39 s relative to sucrose at 100 mM with an onset of 13 ± 2 s and a lingering time of 6 s. Saccharine was shown to activate the bitter receptors TAS2R8, TAS2R31, and TAS2R43, confirming its known off-flavor, whereas addition of cyclamate reduced or blocked this saccharine bitter response. The potential of using this tongue-on-a-chip to bridge the gap with in vitro assays and taste panels is discussed.

## Introduction

The G-protein-coupled taste receptors for sweet, umami, kokumi, and bitter attributes together with alternative sensory systems for saltiness and sourness are naturally expressed in specialized tissues like the sensory epithelium of the tongue.^1^ Receptor cell assays in multiwell plates have been developed,^2^ but there is still a large gap between the outcomes of these in vitro assays and some of the relevant in vivo metrics reported by human taste panels. The microfluidic device presented here with an array of taste receptors mimics the transient exposure to tastants and could potentially bridge this gap.

The sweet and umami taste receptors comprise the G-protein-coupled receptor (GPCR) family TAS1R, and the bitter taste receptors comprise the TAS2R family. In the TAS1R family, the sweet receptor is encoded by the heterodimer of TAS1R2 and TAS1R3, which mediates the sweet sense from most natural sugars as well as noncaloric sweeteners.^3−5^ Glucose sensing is, in addition, mediated via the sodium–glucose transporter SGLT1,^6,7^ so that exposure of taste cells to this natural sugar also triggers parallel pathways different from the sweet GPCR pathway. Bitter taste is mediated by the TAS2R receptor family consisting in humans of 26 functional genes^8^ including the recently added TAS2R2.^9^ Of those 26 bitter receptors, four are still orphan receptors, since no ligand has yet been identified for them, and vice versa, there are still orphan bitters like grapefruit naringin without a functional bitter receptor assay.^10,11^ In the canonical model, the sweet, umami, and bitter taste receptors are all thought to couple to the Gαi type G-protein subunit GαGustducin in complex with Gβ1/3 and Gγ13.^1,12,13^ The current working model on signal transduction states that upon activation of the receptor, the complex of three G-proteins first binds to the receptor and then, after phosphorylation, dissociates from the receptor and initiates a downstream signaling pathway. This downstream signaling pathway of the trimeric complex could result in both a cAMP decrease via Gαgustducin (unblocking the IP_3_R3 channel via PKA^14,15^) and a calcium increase via Gβ1/3-Gγ13 (stimulating PLCβ2 IP_3_ production and opening IP_3_R3),^16^ but this postulated pathway is not working as well as the chimeric G-protein assay for taste receptors (personal communication Dr. M. Behrens). To direct the signaling pathway of the taste receptors toward the more convenient Gαq calcium route for GPCR screening, Gαq chimeras have been developed. Based on the template of Gα15 (human) or Gα16 (rodent), which on their own already couple promiscuously to many different types of receptors,^17^ a chimeric Gα protein of Gα16 with 44 C-terminal residues of gustducin (Gα16GUST44) was designed with good performance for both sweet and most bitter compounds, eliciting an intracellular, metabotropic calcium response upon activation.^18^

**Figure 1:**
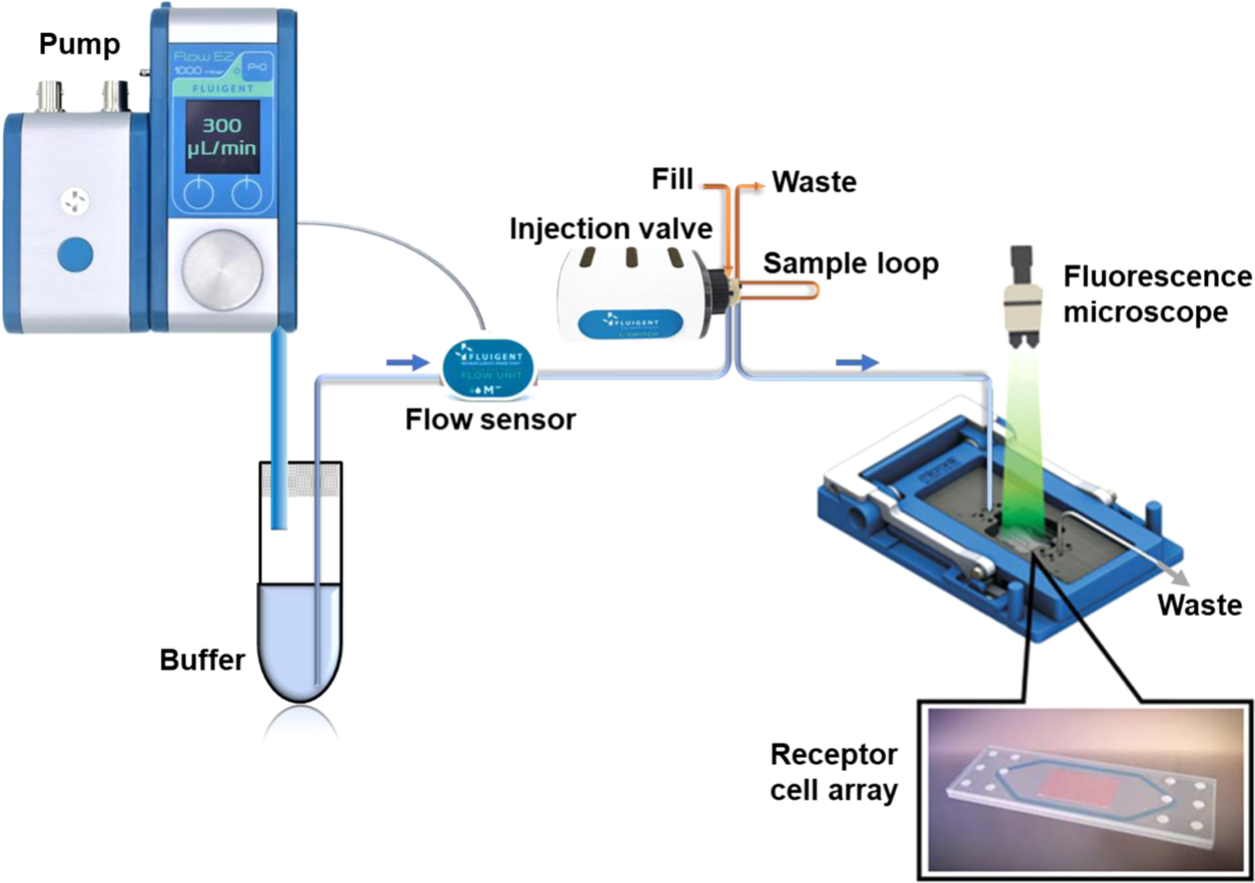
Receptomics setup for the measurement of receptor cell arrays. A constant flow of 300 μL/min is maintained by the air pressure-based pump system (Fluigent) with feedback from a flow sensor unit. Samples are injected using the L-switch valve (Fluigent) and a 300 μL sample loop. The flow cell holder (MicroNit) contained a 50 or 100 μL resealable flow cell (MicroNit). Sample dosing was controlled and calibrated using fluorescein dye, as shown in Supporting Figure 1. This figure is adapted in part with permission from Roelse et al.^23^ Supporting Information and adapted in part with permission from Fluigent (www.fluigent.com).

For this study, we have used HEK293 cells stably expressing the Gα16GUST44 chimera to prepare receptor arrays, which are reverse-transfected into a monolayer of cells adhered to a glass slide. To this end, the slides are first printed with receptor coding plasmid DNA along with the calcium-sensing reporter gene Twitch2B.^19^ The slide or tongue-on-a-chip is then enclosed in a flow cell and connected to a microfluidic system, which ensures a constant flow of assay buffer across the receptor array and allows for sample loop injections into the existing flow, leading to precisely timed periods of ligand exposure (Figure 1). The strength of this specific tongue-on-a-chip application^20^ is the similarity it holds to a human sensory experience of exposure windows to tastant solutions. It also facilitates the parallel assaying of an array of taste receptors versus a series of samples, each tested for a controlled period of time (typically 30–60 s) with ∼5 min intervals to allow sample wash-out and return of the cell calcium levels to baseline. The receptomics application protocol includes several internal controls to allow for an accurate analysis of receptor activation and metrics resembling the kinetics of onset and lingering. To evaluate the operability of this tongue-on-a-chip, we combined a subset of bitter taste receptors and the sweet receptor on one array. This allows for the efficient parallel measurement of response patterns of both sweet and bitter receptors upon stimulation with sweeteners known in some cases for their bitter off-taste and/or lingering. Advantame, for example, is characterized by its quick onset and subsequent prolonged sweet taste lingering,^21^ whereas saccharin and cyclamate are known for their bitter off-tastes. Remarkably, this bitterness is attenuated by combining both, which induces at the molecular level the blocking of the receptors TAS2R43 and TAS2R31.^22^ Here, we aimed as a first step to derive from this tongue-on-a-chip, a set of parameters analogous to a sensory panel and to the endpoint receptor assay experiments. The potential to extend the tongue-on-a-chip platform to other receptors and oral factors and its application with more complex solutions is discussed.

## Materials and Methods

### Chemicals

In this study, the following chemicals were used: adenosine triphosphate ATP (Sigma A6419), chloramphenicol (Duchefa C0113.0100, purity >98%), aristolochic acid (Sigma A5512, ≥90%), picrotoxinin (Sigma P8390, ≥98%), chloropheniramine (Sigma PHR1016, ≥99.8%), quinine (Wako 179–00461, ≥98%), diphenidol (Sanbio 18674–10, ≥ 98%), strychnine (Wako 195–11151, ≥98%), saccharine (Sigma 240931, ≥99%), cyclamate (Sigma 47827, ≥98.9%), sucrose (Duchefa S0809–0925, ≥99.7%), aspartame (Sigma PHR1381, purity unknown), and advantame (Sigma 80054, ≥97%). Some of the abovementioned bitter compounds were toxic and required appropriate safety measures.

### Expression Vectors

DNA arrays for reverse transfection were prepared and printed as previously described in Roelse et al.^23^ The genes encoding bitter TAS2R3, TAS2R8, TAS2R14, TAS2R31, TAS2R43, and TAS2R46 (see Roelse et al.^24^ for sequence information) were obtained from genomic DNA of HEK293 cells by PCR amplification and were cloned into pcDNA3 containing the N-terminal sstr3 tag (gift from Dr. Wolfgang Meyerhof, German Institute of Human Nutrition Potsdam-Rehbrücke, Germany). The expression vectors encoding TAS1R2 and TAS1R3 originated from the Japanese group of Abe,^25^ and TAS1R1 was obtained from Genscript code OHu15305D. Plasmid encoding Twitch2B in pcDNA3 was obtained from Oliver Griesbeck (Addgene plasmid # 49531).

### Receptomics Assay

DNA-printed receptor arrays on a glass slide were reverse-transfected into a monolayer of HEK293 cells that stably expressed Gα16GUST44 (plasmid provided by Dr. Takashi Ueda, Nagoya City University, Nagoya, Japan). Arrays were incubated for 48 h at 37 °C with 5% CO_2_. For sweet receptor experiments, the cell arrays were first preincubated for 2 h in Low Glucose DMEM supplemented with pyruvate (Gibco 11880028). After this preincubation, the arrays were taken from the incubator, washed three times, and incubated in glucose-free assay buffer (NaCl 115 mM, KCl 5 mM, CaCl_2_ 2 mM, HEPES 10 mM, and sodium pyruvate 1 mM at pH 7.4) for at least 1 h prior to performing the measurements. Each fluidic sample series was injected into an ∼50 or 100 μL flow cell in a flow cell holder (Micronit Microfluidics B.V., Fluidic Connect PRO Chip Holder). The flow cell was connected to the Fluigent pump setup, as shown in Figure 1, unless otherwise mentioned. This setup was composed of a compact pressure source, LineUp LINK, LineUp FlowEZ pump, Flow Unit L, and the L-Switch injection valve. In cell array experiments, the assay buffer was set to a continuous flow of 300 μL/min across the array, and sample injections were performed with an injection volume of 300 μL. The arrays were imaged with a Leica fluorescent stereo microscope (Leica M205FA as previously described^24^). For Figure 3C,D, a newly designed microscope setup was used, i.e., a custom-made dual-channel microscope (DCM) developed by PhenoVation (www.phenovation.com). This DCM microscope was dedicated to FRET measurements, and images at two different wavelengths (CFP at 480/36 nm and YFP at 535/25 nm) were simultaneously captured by the same sensor (12 MP CMOS) of the camera.

### Data Analysis

The analysis of data from the receptor cell arrays was performed as described in Wehrens et al.^26^ using our ReceptomX software. In short, FRET images for the CFP and YFP channels were recorded and converted, using the CellProfiler software package, to raw CFP and YFP intensity values. Spots with less than 15 fluorescent pixels and spot types with fewer than 5 replicates were removed from the data analysis. After smoothing and interpolation to remove the differences in timing between the CFP and YFP measurements, spot signals were calculated as the ratios of the CFP and YFP values. These signal peak heights associated with individual injections were calculated as the difference between the start and maximum ratio values in the spot signal (iRatio).

### Statistics

The iRatio values, after log scaling, were used in a mixed model with the injection type as a fixed variable and the spot number as a random variable. Results, presented in Figure 4C–H, consist of treatment-versus-control contrasts, which are noted below the plots. This leads to coefficients that should be interpreted as multiplicative effects: a value of 1.1 should be interpreted as a 10% increase in response compared with the reference. A value of 1.0 indicates no difference to the reference. Each plot in Figure 4C–H represents a separate treatment-versus-control contrast for each group of spots with a particular receptor type. The estimates are plotted with a 95% confidence interval. The mixed model used for these contrasts is described in Wehrens et al.^26,27^ Significant results, not including 1 in the confidence interval, are highlighted in red.

## Results

### Sample Exposure Control Parameters

The receptor cell array expressing recombinant sweet, umami, and bitter receptor genes was mounted in a microfluidic system using an air pressure-based pump system (Fluigent), which uses a flow sensor’s feedback to maintain constant flow rates (Figure 1). The injection system based on an injection loop of fixed volume allows for a controlled exposure duration of samples to the array based on the chosen flow rate and flow cell volume. Table 1 summarizes this, based on triple measurements with a fluorescein dye in an empty flow cell, to determine the different peak shapes (rise, duration (width), fall) corresponding to the injection variables. The most commonly used settings in our receptomics experiments are marked in bold in Table 1. The corresponding fluorescein peaks of the injections, as shown in Supporting Figure S1, display a high degree of reproducibility between subsequent exposure peaks with a variability of <1 s for the full width at half-maximum (fwhm).

**Table 1:** Effect of Flow Cell Volume, Injection Volume, and Flow Speed on Peak Width, Rise, and Fall as Measured Using Fluorescein in an Empty Flow Cell with a Flow of in Ultrapure Water

flow cell volume in μL	injection volume in μL	flow speed in μL/min	theoretical exposure time in s^a^	fwhm^b^ in s	maximum rise/s in %^c^	maximum fall/s in %^c^
50	300	100	180	185 (±1)	2.4	–1.3
50	300	300	60	60	5.0	–3.3
50	300	600	30	30	10.3	–6.0
50	300	900	20	21	15.8	–8.3
50	1000	100	600	564	2.4	–0.5
50	1000	300	200	182	5.4	–1.4
50	1000	600	100	91	11.7	–2.6
50	1000	900	66	61	15.9	–3.5
100	300	100	180	186 (±1)	2.1	–1.3
100^d^	300^d^	300^d^	60^d^	59^d^	4.5^d^	–3.4^d^
100	300	600	30	31	8.9	–6.8
100	300	900	20	20	10.9	–8.2
100	1000	100	600	566	2.1	–0.6
100	1000	300	200	182	4.3	–1.4
100	1000	600	100	91	7.2	–2.6
100	1000	900	66	61	11.0	–3.5

a Theoretical exposure based on flow speed and injection volume without sample diffusion, laminar mixing, and multisport averaging.

b Full width at half-maximum of the fluorescein peak.

c Maximum rise and fall rate of the fluorescein curve.

d The settings used in tongue-on-a-chip measurements.

Table 1 shows both the theoretical exposure time in seconds when no peak broadening due to sample diffusion, laminar mixing, and multispot averaging would occur, as well as the actual recorded values that include these effects. The spread of spots on the array differs by several seconds in exposure start and end time depending on their position on the array and could introduce a small offset in the rise and fall flank. In practice, the peak shape is, therefore, an S-curved upward slope, a plateau maximum, and an S-curved downward slope. This is a constant design feature affecting all samples equally, but the slopes were steeper at higher flows, as shown by a faster rise and fall rate of the fluorescein curve. The fwhm corresponded well to the theoretical peak width for the injection volume except for the 1000 μL injection volume, which was 10% less in fwhm, suggesting that possibly the 1 mL sample loop might have had a smaller internal volume or was shorter in length than specified.

Independent of the injection volume or flow speed, the fluorescein signal rise was consistently steeper than the signal drop due to the fact that the signal rise represents the filling of the flow cell with fluorescein dye, which has a shorter diffusion and mixing time than the drop that follows.

### Taste Receptor Gene Dose Optimization

In reverse-transfected receptor arrays, the expression level of a gene of interest is determined by the portion of coding plasmid DNA in the total DNA content of the print mixture.^23^ We have determined previously that the gene dose of the calcium sensor protein is crucial for optimal calcium sensing, since high expression levels lead to calcium buffering.^24^ A similar, though mechanistically different, situation also applies to receptor gene doses. Therefore, we also aimed to optimize the gene dose of the taste receptors to yield the highest calcium signals upon ligand activations.

Arrays were printed with variable receptor gene doses of bitter and sweet taste receptors. The total DNA content in the print solution was kept constant at 75 ng/μL DNA using empty vector DNA to supplement the total DNA. For the sweet taste receptor heterodimer, the optimal gene dose and gene ratio were determined using dedicated arrays with a gene dose titration of either gene ranging from 16.7 ng/μL down to 1 ng/μL gene dose. Higher gene doses were tested previously and were found to negatively affect the sweet receptor responses (not shown). The sweet receptor was activated using a concentration series of aspartame, as shown in Table S1 and Figure S2. Table S1 summarizes the sweet receptor response to 2.5 mM aspartame with different gene doses and gene ratios and shows an optimum response at 16.7 ng/μL for TAS1R2 and 8.3–16.7 ng/ μL for TAS1R3. Similar data were obtained with saccharine (not shown).

Table S2 and Figure S3 show the effect of gene dose for the bitter taste receptors used in this study and their optimum response to their ligands at gene dose levels ranging from 67 to 2 ng/μL. Table S2 shows the maximum iRatio values of a representative ligand for each receptor. The maximum response is different for each bitter taste receptor with the highest dose of 67 ng/μL, yielding optimal responses for TAS2R8, TAS2R14, TAS2R43, and TAS2R46 and lower doses of 16.7 ng/μL for TAS2R3 and 8.3 ng/μL for TAS2R31.

### Sweet Receptor Response EC50

**Figure 2:**
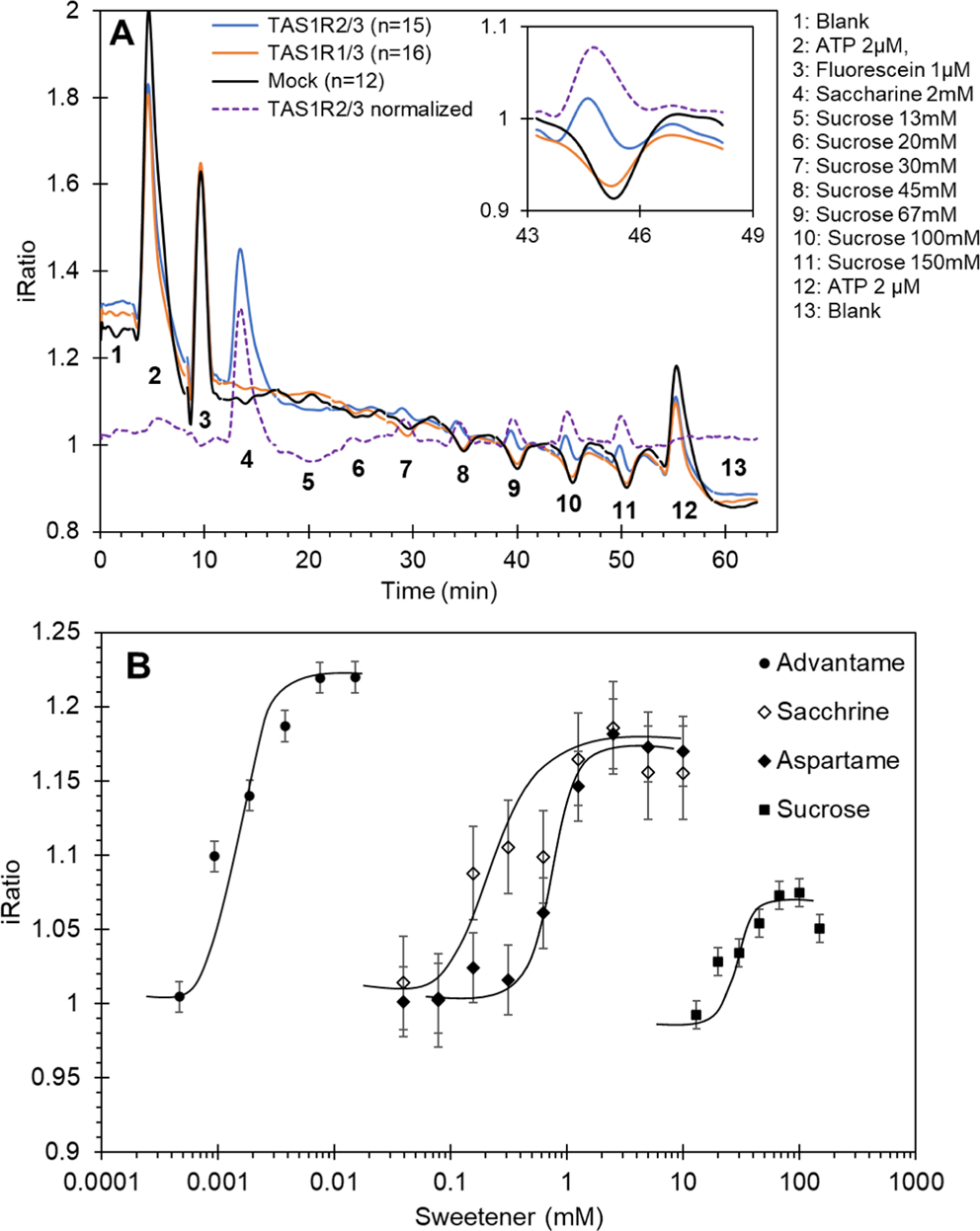
Concentration–response relationship of the sweet receptor TAS1R2/R3. (A) Sucrose dose–response series. The traces for the sweet receptor (blue), umami receptor (orange), and mock (black) are shown. The dotted line is the sweet response normalized against the umami signal. The inset shows the response of 100 mM sucrose enlarged. (B) Dose–response curves of TAS1R2/R3 of sweeteners and sucrose. EC50 values were estimated as ∼0.0015 mM for advantame, 0.3 mM for saccharine, 0.625 mM for aspartame, and 35 mM for sucrose. The dose–response curves were measured on four different arrays. The spot replication level was *n* = 15 for advantame, *n* = 11 for saccharine, *n* = 13 for aspartame, and *n* = 11 for sucrose.

**Figure 3:**
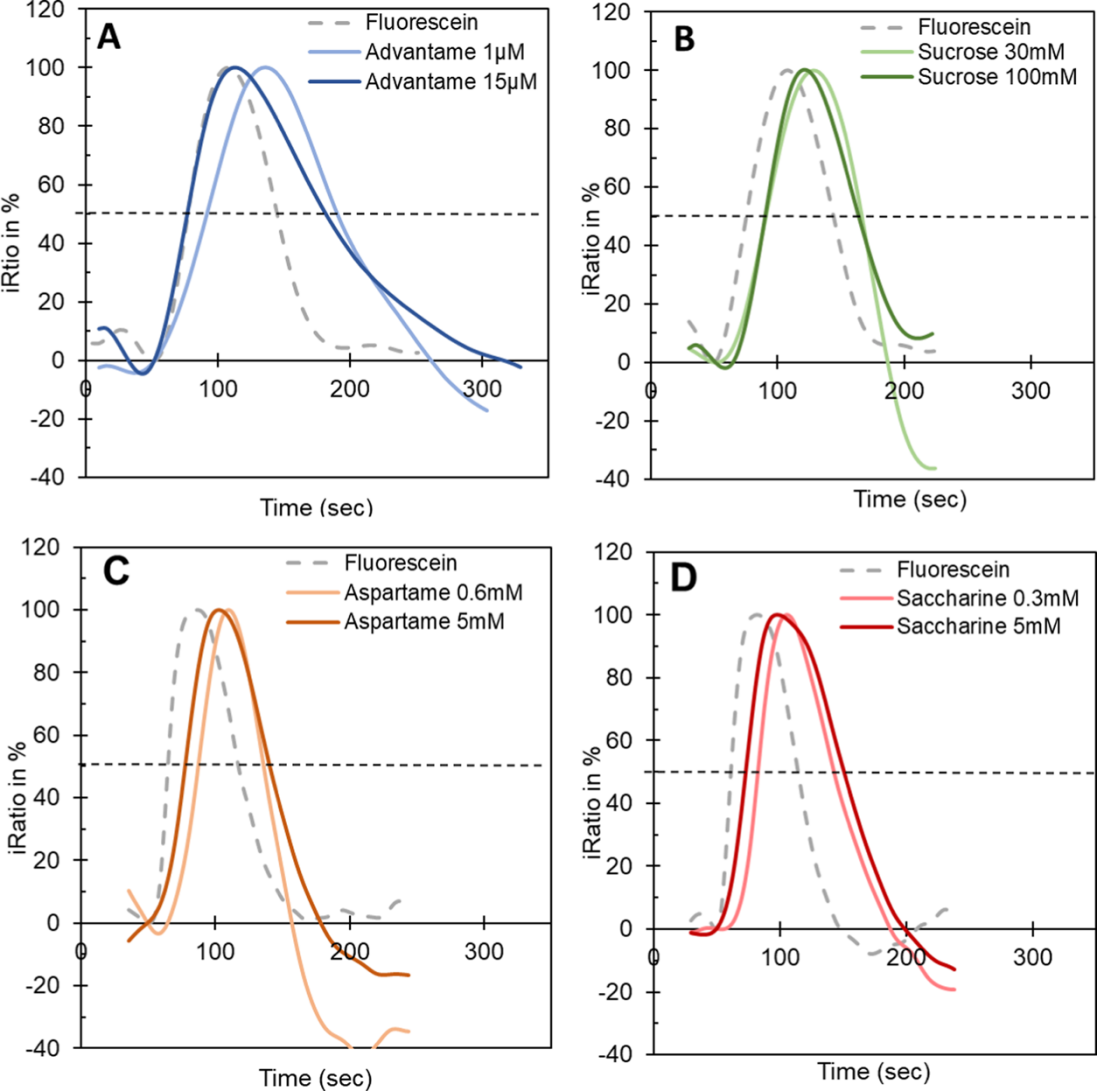
Sweet receptor response calibrated for the ligand concentration relative to a fluorescein injection (dashed line). The sweet receptor response profiles to advantame (A), sucrose (B), aspartame (C), and saccharine (D) from Figure 2 are plotted. For each agonist, the response at the respective EC50 concentrations and at the maximum dose is plotted. All curves are synchronized to start at *T* = 50 s, and all responses are normalized to 100%. The dotted line indicates the 50% response value used to determine the fwhm (Table 2).

Having determined the optimal gene doses of the heterodimer sweet receptor, 16.7 ng/ μL for both TAS1 genes and various optimized gene doses for the bitter receptors, arrays were designed that included the umami receptor in the same gene dose as the sweet receptor, a mock (no recombinant receptor DNA added), and a sensor control YC- (fixed FRET ratio to monitor sample effects on the fluorescence readout). Four separate dose–response curves were prepared for advantame, aspartame, saccharine, and sucrose, as shown in Figure 2B. The mock and umami traces are displayed as controls in the dose–response plot of sucrose in Figure 2A. In contrast to the sweetener dose–response series, there was a considerable iRatio dip in the umami and mock curves for sucrose, while the sweet receptor curve showed iRatio peaks, which corresponded to an increase in intracellular calcium. The correct(ed) signal dynamic of the sweet receptor response to sucrose was, therefore, obtained by normalization against the umami signal (dotted line). This normalized signal was used for the values of the dose–response signals and the kinetic curves of Figure 3B. The half maximal effective concentrations (EC50) for the dose–response series of the sweeteners were determined from Figure 2B to be ∼1.5 μM for advantame, 0.3 mM for saccharine, 0.625 mM for aspartame, and 35 mM for sucrose; see also Table 3.

### Sweet Receptor Onset and Lingering

The samples Figure 3A,B were prepared with the microfluidic system of Figure 1, while the samples in Figure 3C,D were prepared using an automated sample injection system (Waters 2795) with a continuous flow of 300 μL/min and an injection volume of 300 μL. The sample reference peaks for both systems are provided by the fluorescein injections in Figure 3. The flow rates of the Waters (Figure 3C,D) and Fluigent (Figure 3A,B) microfluidic injection systems were not calibrated, which may explain the differences in exposure durations between the systems. However, the kinetic metrics are always expressed relative to the fluorescein injection response profile from the same experiment. Furthermore, we do not expect a difference in the Δfwhm relative to fluorescein due to a difference in flow rate, as the Δfwhm is a factor intrinsic to the cellular activation/deactivation kinetics and not of the exposure duration or flow rate. The responses were all fitted to a percentage scale, where 100% was the maximum response, and the injection times were synchronized to *T* = 50 s. The figures show the superposition of (i) the fluorescein injection, (ii) the response to an ∼EC50 concentration of the sweetener, and (iii) a concentration (3–15× higher), causing a maximum response. The calcium response of the cells to the sweeteners and sucrose increased in parallel to the fluorescein injection but generally reached a maximum later than the maximum of the fluorescein sample. We propose that the metric describing the tongue-on-a-chip peak maximum delay could be a reliable (partial) equivalent of what is called the taste onset or time to peak sweetness in human sensory assessments when it is expressed relative to the fluorescein peak maximum. For this metric to be comparable and most accurate, we propose that the sample exposure time should always be short to avoid broad maxima and should be similar across experiments to allow valid comparisons. Alternatively, it could be based on the 90% maximum point, as explained in Figure S4. We found that relative to the high-concentration samples, it generally took the lower EC50 doses longer to reach their maxima (Table 2). In contrast, the onset time of 5 ± 2 s for advantame at the high 15 μM dose stood out from the other sweeteners, as it was almost instantaneous and coincided with the fluorescein exposure.

**Table 2:** Peak Kinetics of Sweet Receptor Responses to Sweeteners Relative to the Fluorescein Reference in the Same Set of Sweet Receptor Spots

sample	dose	onset in s^a^	fwhm in s^b^	Δfwhm lingering in s^c^	maximum rise/s in %^d^	maximum fall/s in %^d^
sucrose	30 mM	20 ± 2	75	6	2.2 (83%)	–2.5 (124%)
	100 mM	13 ± 2	75	6	2.6 (99%)	–1.6 (77%)
fluorescein		0	69	0	2.6 (100%)	–2.0 (100%)
advantame	1 μM	28 ± 2	102	34	1.7 (44%)	–1.2 (59%)
	15 μM	5 ± 2	107	39	2.3 (77%)	–0.9 (42%)
fluorescein		0	68	0	3.0 (100%)	–2.1 (100%)
aspartame	0.6 mM	24 ± 1	52	none	3.4 (48%)	–2.7 (106%)
	5 mM	18 ± 1	65	12	3.4 (48%)	–1.9 (73%)
fluorescein		0	53	0	6.9 (100%)	–2.6 (100%)
saccharine	0.3 mM	24 ± 1	60	7	4.2 (64%)	–1.8 (71%)
	5 mM	16 ± 1	78	25	3.6 (56%)	–1.6 (64%)
fluorescein		0	53	0	6.5 (100%)	–2.5 (100%)

a Onset or peak maximum delay compared to the fluorescein reference of the same series; see also onset 1 in Figure S4.

b Full width at half-maximum of the fluorescein peak.

c fwhm increase compared to fluorescein reference of the same series.

d Maximum rise and fall rate of the curves in %, in brackets the relative response rise or fall compared to the fluorescein reference of the same series.

Next to the onset, the width of a response peak can be determined reliably from the fwhm measurement. This is the half-maximum time interval between the two slopes of the response peak, as shown in the kinetic plots in Figure 3. The time difference, Δfwhm, to the fluorescein reference we propose as the closest and most accurate metric to describe what is known as “lingering” in human sensorial assessments (delayed curve drop). This Δfwhm lingering is considerably longer for advantame compared to the other sweeteners and stands out by having a lingering time of 34–39 s compared to sucrose with 6 s for both concentrations. The sweeteners aspartame and saccharine show lingering also, but the maximum dose has a much longer lingering effect (12 and 25 s.) compared to the EC50 dose (none and 7 s).

The response rise rate and the recovery fall rate back to baseline at half-maximum offer independent metrics that could potentially also predict both onset and lingering accurately (Table 2). The receptor calcium maximum rise rate relative to fluorescein at both high and low concentrations is less for the sweeteners than for sucrose, similar to onset differences (Table 2). The fall rates at both concentrations are similar to lingering differences between sweeteners and sucrose. The value of these metrics needs to be assessed next to panel ratings and be based on identical samples in follow-up studies to validate which metric or converted metric can best be used. Table 3 is an illustration of how panel ratings are also often variable in their reported outcomes.

**Table 3:** Sweetness Kinetics in the Tongue-on-a-Chip Assay Compared to Reference Cell-Based Assays and Sensory Panel Attributes

	EC50	EC50	taste threshold	onset	onset, time to peak sweetness	lingering	lingering
	tongue-on-a-chip^a^	multiwell assay	sensory panel	tongue-on-a-chip^b^	sensory panel^c^	tongue-on-a-chip^d^	sensory panel^e^
sucrose	35 mM	∼60 mM^3^	∼20 mM^3^	20 s ± 2 (30 mM)	9.7 s^33^	6 s (30 mM, 100 mM)	6.8 AUC^33^
		26 mM^40^	7–10 mM^37^	13 s ± 2 (100 mM)	8 s^34^ (292 mM)		28.2 AUC^34^ (292 mM)
			5.5 mM^41^		1.09 s ± 1.81^21^ (146 mM)		119 s ± 53^21^ (146 mM)
advantame	1.5 μM			28 s ± 2 (1 μM)	5.67 s ± 2.83^21^ (6.5 μM)	34 s (1 μM)	139 s ± 75^21^ (6.5 μM)
				5 s ± 2 (15 μM)		39 s (15 μM)	
aspartame	0.625 mM	∼1 mM^3^	∼1 mM^3^	24 s ± 1 (0.6 mM)	9.6 s^33^ (3.8 mM)	12 s (5 mM)	10.9 AUC^33^ (3.8 mM)
		0.75 M ± 0.11^42^	22.5 μM	18 s ± 1 (5 mM)	8 s^34^ (2.8 mM)		33.8 AUC^34^ (2.8 mM)
		0.145 mM^43^			4.10 s ± 3.07^21^ (0.8 mM)		125 s ± 53^21^ (0.8 mM)
saccharine	0.3 mM	0.19 mM ± 0.07^42^	14.7 μM^41^	24 s ± 1 (0.3 mM)	1.27 s ± 2.21^21^ (1.2 mM)	7 s (0.3 mM)	146 s ± 62^21^ (1.2 mM)
				16 s ± 1 (5 mM)		12 s (5 mM)	

a EC50 values determined in Figure 2B.

b Onset value from Table 2 with concentration in brackets.

c Values obtained from various studies corresponding to concentrations in brackets.

d Lingering values from Table 2 with concentration in brackets.

e Values obtained from various studies with lingering expressed in AUC (area under the curve) or s.

### Off-Flavor Bitterness of Sweeteners

Arrays with optimal gene doses for the sweet receptor TAS1R2/3, bitter receptors TAS2R3, TAS2R8, TAS2R14, TAS2R31, TAS2R43, TAS2R46, a mock with only the calcium sensor gene, and a sensor control YC- (fixed FRET ratio to monitor sample effects on the fluorescence readout), were used in experiments to demonstrate the bitter off-flavor of sweeteners and how sweeteners interact at the receptor level. The set of bitter receptors was chosen to include the known saccharine receptors TAS2R31 and TAS2R43,^22^ one low-affinity saccharin receptor, TAS2R8,^28^ and three bitter receptors with no link to saccharine, TAS2R3, TAS2R14, and TAS2R46. The set did not include all bitter receptors in order to get higher quality data from the replications than what is possible on a single chip. The array was exposed to samples of saccharine and cyclamate both pure and as a mixture following the publication of Behrens et al.,^22^ but now in the tongue-on-a-chip setting. A high stimulus of 10 mM saccharine and a high blocking dose of 20 mM cyclamate were chosen to ensure adequate activation and blocking of the bitter receptors. Cyclamate on its own can activate the bitter receptors, TAS2R1 and TAS2R38, at threshold concentrations of 30 mM.^22^ However, such high concentrations of cyclamate are not common in food samples and therefore not relevant in considering bitter off-flavor. These high concentrations of cyclamate and the corresponding bitter receptors were not included in the array for that reason.

**Figure 4:**
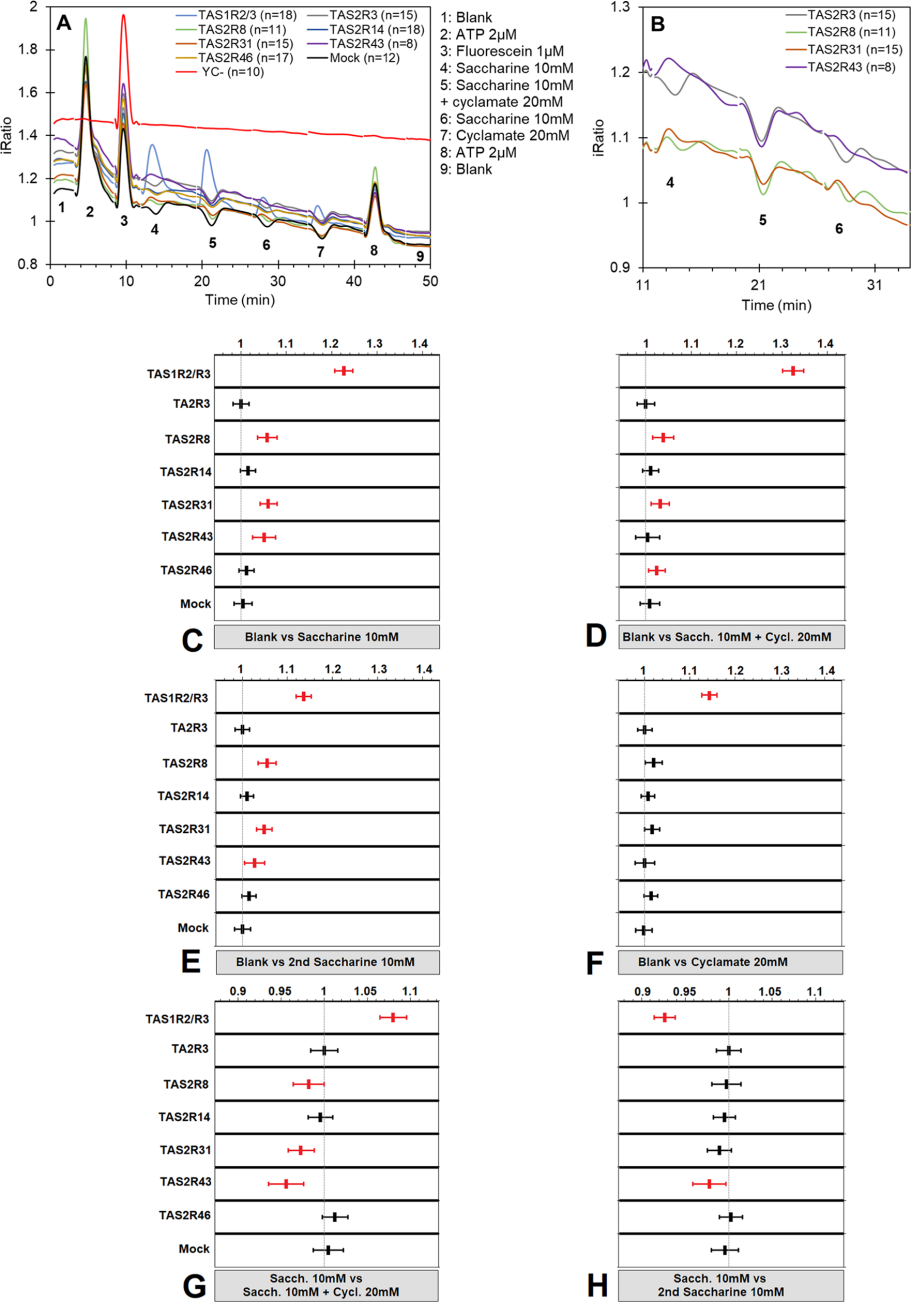
Blocking of multiple saccharine bitter responses by cyclamate in sequential assays. (A) iRatio plots for all receptors on the array including the sensor control YC. A series of nine samples was injected, as indicated by the numbers in the plots. (B) Zoom of the iRatio plot for TAS2R8, TAS2R31, TAS2R43, and TAS2R3 for injections 4, 5, and 6. (C–H) Estimates and 95% confidence intervals for the control-vs-treatment contrasts for each of the receptor types on the array. The top panels C/D/E/F show the contrast of the blank versus the sample. The lower panel G shows the contrast between the first saccharine exposure versus the mixture of saccharine with cyclamate. Panel H shows the contrast between saccharine and the second saccharine exposure. Significant results, not overlapping with 1 in the interval, are marked red. Sacch = saccharine and Cycl = cyclamate.

Figure 4A shows the average raw response traces of the sweet receptor, six bitter receptors, the mock, and the YC- control. The high concentrations of the sweetener did not affect the FRET ratio measurement because no change in iRatio output from the sensor control, YC-, was observed. The TAS2R3, TAS2R14, TAS2R46, and mock traces show a small dip during exposure to these high sweetener concentrations. In Figure 4B, the response traces for injections 4, 5, and 6 are enlarged for TAS2R8, TAS2R31, and TAS2R43 to show more clearly the response to saccharine relative to the nonresponsive TAS2R3, which represents the baseline for the response estimates in Figure 4C–H. The signals in Figure 4C–H provide the statistical control-vs-treatment contrasts for the complete set of receptors. These include contrasts of the blank versus samples 4, 5, 6, and 7 in Figure 4C–F, respectively, and the intersample comparisons between injections 4 and 5 in Figure 4G and 4 and 6 in Figure 4H. The estimates of these contrasts are plotted as a 95% confidence interval. Significant results, which do not overlap with 1 in the confidence interval, are highlighted in red.^26^

Figure 4C shows the significant responses to 10 mM saccharine for TAS2R8, TAS2R31, and TAS2R43. These responses are fully (TAS2R43) or partially blocked in combination with 20 mM cyclamate because, as shown in Figure 4D, the bitter responses were reduced or had returned to 1 (TAS2R43). The blocking effect can be calculated by the % of value reduction between Figure 4C and D. This resulted in a 33% signal reduction for TAS2R8, a 48% signal reduction for TAS2R31, and a 92% signal reduction for TAS2R43. The significance of the blocking effect is shown in Figure 4G with the intersample comparison between 10 mM saccharine and 10 mM saccharine supplemented with 20 mM cyclamate. The second exposure of 10 mM saccharine (injection 6) in Figure 4E reproduces the significant activation of TAS2R8, TAS2R31, and TAS2R43, which indicates that the blocking mode of cyclamate is reversible. On its own, cyclamate did not activate this set of bitter receptors, as shown in Figure 4F.

There is a strong activation of the sweet receptor by 10 mM saccharine in Figure 4C, and this activation is enhanced by the combination of 10 mM saccharine and 20 mM cyclamate in Figure 4D. Subsequently, the repeated 10 mM saccharine injection in Figure 4E and subsequent injection of 20 mM cyclamate in Figure 4F resulted in lower sweet receptor response peaks. This can be explained by a process of receptor desensitization, which is often observed in repeated injections with high ligand concentrations. This desensitization is also observed for TAS2R43 when comparing the first and last response to 10 mM saccharine (injections 4 and 6) in Figure 4H, where the TAS2R43 bitter response to saccharine has significantly declined. This desensitization can be explained by the sensitivity of TAS2R43 for saccharine, which is, at 10 mM, at the maximum of its dose–response curve as published previously by Behrens et al.^22^

## Discussion

This study aimed to establish how the metrics provided by the tongue-on-a-chip receptomics flow cell setup could potentially serve the flavor research community with an in vitro tool to measure sensory parameters that are otherwise more difficult, less reproducible, or costly to obtain in vivo with human taste panels.^29,30^ We have shown that the tongue-on-a-chip platform, with taste receptors for sweet and bitter, generated metrics analogous to sensory evaluations such as taste threshold, onset, and lingering. We also demonstrated how it correctly identified sensory interactions (antagonisms) of tastant combinations at the receptor level that were earlier obtained with conventional cell assays using multiwell plates.^2^ Currently, tongue-on-a-chip is limited to 3 of the 5 basic tastes. The salty and sour tastes have been suggested to be mediated by the ENaC and OTOP1 ion channels^31,32^ and, in order to add these tastes to the tongue-on-a-chip, adjustments should be made at the level of the sensors (ions, membrane potential, pH) and/or at the level of the cell line to tolerate sour and salty samples. We expect that building further on this tongue-on-a-chip platform can establish efficient methods to study the taste of complex foods and beverages and include modulating effects of mucous layers containing saliva proteins, enzymes, and the oral microbiome. To reliably predict taste panel ratings, it will be necessary to take the data through machine learning software to adjust the outcomes relative to panel ratings for specific food/beverage categories.

### EC50 and Taste Thresholds

The tongue-on-a-chip platform is based on a microfluidic system of injected samples that are passed over the array by means of a constant flow of assay buffer. The system was used to generate several dose–response curves of sweeteners with the sweet receptor. Each dose–response curve is based on a single experiment with a series of injections with increasing concentrations of the sweetener. For 11–15 replicated spots, individual response traces were measured, and the averages were plotted. This method of determining the concentration–response relationship of a receptor is relatively quick and uses much less sample, transfection reagents, buffers, and/or dyes than a multiwell plate system would need for a similar experiment. The fluidic nature of the setup also raised the interesting possibility of extracting results that parallel metrics generated by taste panels such as onset, lingering, and off-taste.^21,33,34^ The spot replicas can be considered true biological replicates for transfection, since each spot represents a separate reverse-transfected event on the array. In the experiments shown, the spots were exposed to the same sample only once. Two to three technical replicates could further improve the data analysis and also correct for any desensitization after repeated challenges. Based on the current data set without this correction, the curve maxima may be somewhat reduced by the repeated challenges, and this may have slightly biased the estimated EC50 to a lower value. Yet, even with this data set, the estimated EC50 values were found to be very close to reported values based on multiwell plate measurements, as shown in Table 3. For advantame, the EC50 value of 1.5 μM is over 20.000× lower than sucrose,^35,36^ which is in line with the known relative sweetness of advantame to sucrose as determined by taste panel assays.^21^ The taste thresholds might be deduced from the lowest concentration (e.g., EC10), yielding a significant response value in such concentration–response curves. We noticed that there is a large variability in the taste thresholds reported by panels in the literature (Table 3). Most likely this is due to variation in parameters like sample size, in-mouth incubation time, age, and genetics of the panelists.^37^ It emphasizes that a consistent in vitro method may be an advantage.

### Onset and Lingering

The ligand exposure time was calibrated with a fluorescein dye injection that is part of each tongue-on-a-chip experiment and allows modeling of both the onset and lingering of receptor calcium responses in terms of a Δ*t*ime delay between the two traces. This was visualized by the superposition of the fluorescein peak and the subsequent sweetener calcium response peaks in the same experiment. We observed a response peak delay for most sweetener samples and proposed to correlate this value with the time to peak sweetness or taste onset of the sensory panels. Published onset values of human panelists are shown in Table 3, with their respective test concentrations in brackets. These test concentrations are an important factor to consider, since the higher concentrations show a shorter onset in the tongue-on-a-chip measurements. The time to maximum taste intensity is usually recorded after 5 s of in-mouth incubation and swallowing of the sweetener solutions. Depending on the measurement methods, the sensory data are again highly variable between studies. Therefore, the intersample differences are best compared within the same sensory study to minimize this variability. The most complete study of sensory parameters, with a large set of sweeteners including the ones used in this study, was found in the study of Karl et al.,^21^ and these data points are included for convenience in bold in Table 3. The sensory onset values between sucrose and saccharine were relatively similar, and so were the onset values in the tongue-on-a-chip. Advantame has a very long onset, as reported in Karl’s sensory study, while here, the tongue-on-a-chip revealed a long onset at the lower concentration and a very short onset at the high concentration of advantame. There is room to speculate about the concentration effect of the sweetener with respect to the onset, since these sensory evaluations did not include different concentrations. However, comparing the absolute tongue-on-a-chip onset values to the sensory data of Karl et al.,^21^ there is a considerably longer onset value when measured using the tongue-on-a-chip system. This could be due to the definition of onset in the tongue-on-a-chip experiments, where the slope gradient may prove to be a better metric. Or it may be due to differences in sample incubation or exposure time, sweetener concentration, or the signaling mechanisms between taste cells and HEK293 cells. Therefore, the absolute onset values may not be representative of the human sensory metrics and could be shifted by a certain value (x seconds). The relative onset values of the tongue-on-a-chip, however, may still predict the human taste onset between samples. Further studies parallel to human sensory trials should therefore be performed to establish the best correlation.

Taste lingering has been quantified in seconds or as the area under the curve (AUC) in sensorial plots (Table 3). Similar to taste onset, the sweetener concentration as well as the measurement methods may influence these values. In in vitro calcium response assays, the AUC is not directly comparable between sweeteners because some sweeteners are known to elicit stronger calcium responses than others. We proposed instead to measure the lingering as the Δfwhm of the receptor response compared to the fluorescein control peak. This Δfwhm lingering time was found to be short for sucrose and considerably longer for the other sweeteners, with advantame being the longest lingering sweetener. Similarly, in the sensorial panel data, the sucrose lingering was also lowest scoring of the sweeteners, while advantame and saccharine scored relatively high.^21^ The absolute lingering times reported by the sensorial panels are significantly longer than those for the tongue-on-a-chip. This may be explained by other factors that may contribute to the lingering of the taste such as saliva composition, saliva flow, etc. Overall, the relative lingering observations of the tongue-on-a-chip are similar to the reported sensorial properties by Karl et al.,^21^ with the exception of saccharine. Saccharine scored higher for lingering in the taste panel assay, while with the tongue-on-a-chip, the saccharine sweetener scored a lingering value comparable to that of aspartame. If this result can be replicated independently, it would suggest that the complexity of the human taste kinetics is not fully explained by the tongue-on-a-chip model. Therefore, again, further studies should be performed to understand the lingering receptor activation in relation to taste lingering, as experienced by a taste panel.

Both the onset and lingering metrics originate from a complex process of taste perception in which the molecular basis that governs these taste attributes is not fully explained yet.^21,38^ We propose here to match the signaling behavior of sweet receptor expressing cells in vitro to this complex system of taste perception based on the analogies in metrics that we observed. Machine learning algorithms may be developed to more closely match the results from taste panels with the tongue-on-a-chip, but whether it would or could hold in all cases needs to be explored further.

### Blocking and Enhancing Effects

The repeated sequential challenge of a tongue-on-a-chip with different samples allows the measurement of blocking and enhancing taste modulation effects by molecules interacting at the receptor level. A well-known example is the moderately bitter off-taste of saccharine.^21^ Bitterness was shown to be induced by the activation of bitter taste receptors TAS2R31 and TAS2R43 and blocked by a second sweetener cyclamate as published by Behrens et al.^22^ We aimed to reproduce these results with the tongue-on-a-chip platform using relatively high levels of the sweeteners to observe clear activation and blocking effects as described.^21^ The tested level of saccharine (10 mM) was comparable to product concentrations of saccharine, which may vary from 2 mM for soft drinks to 3000 mg/kg (=16 mM), for e.g., chewing gum (source: www.fao.org/3/Y0474S/y0474s5t.htm). Cyclamate was applied as a blocker at 20 mM, which is relatively high, since product concentrations do not usually exceed 15 mM (source: www.fao.org/3/Y0474S/y0474s2u.htm). Cyclamate itself can also activate the bitter receptors TAS2R1 and TAS2R38 at threshold concentrations of 30 mM,^22^ but, since this is not a realistic concentration in food products, the test for cyclamate bitterness was not included in this experiment. The results show a blocking effect of 10 mM saccharine by 20 mM cyclamate not only for the bitter receptors TAS2R31 (48% signal reduction) and TAS2R43 (92% signal reduction) but also for TAS2R8 (33% signal reduction). The results for TAS2R31 and TAS2R43 are in line with the results of Behrens,^22^ where TAS2R8 was mentioned as a low-affinity target for saccharine, but its activation and subsequent blocking of cyclamate could not be confirmed in their experiments. Here, we show that TAS2R8 is indeed also involved in the bitter off-taste perception and is surprisingly blocked to some extent by cyclamate.

## Concluding Remarks

The combination of a microfluidic assay with an array of sweet and bitter taste receptors was shown to emulate the functionality of a tongue-on-a-chip, generating sensory parameters analogous to taste panels including those for off-taste, taste threshold, onset, lingering, and blocking interactions of pure and mixed tastant samples. However, while there is not an exact similarity with panel ratings, the tongue-on-a-chip kinetic parameters could explain, at least in part, what panels rate based on conventional methods.^38^ Larger data sets are needed to assess the benefits and limitations of the platform and to evaluate the power of machine learning for more accurate predictions. Our goal is to further expand the tongue-on-a-chip with the other relevant receptors (umami, kokumi, salty, sour, pungent, hot, cool, etc.) and to implement the platform with more complex food extracts. A current obstacle with crude food extracts is, however, the host cell response triggered by naturally occurring metabolites, such as purines. HEK293 cells naturally express receptors that are triggered by these compounds and in turn deliver erroneously high values. However, there are potential ways to tackle this issue both with blockers and by editing out the problematic receptor genes. Fine-tuning the real-world behavior and performance of this in vitro tongue-on-a-chip may also require introducing elements of the mucosal pellicle that adsorbs and processes biomolecules, which, by some, is regarded as a key component of oral physiology.^39^ Expression of mucosal proteins next to taste receptors may represent a first step in this process.

## Supporting Information Available

Two tables on gene dose optimization: the sweet receptor (Table S1) and the bitter receptors (Table S2); four supporting figures: system calibration sequence with fluorescein dye (Figure S1); dose–response curves for gene dose optimization of the sweet receptor (Figure S2); bitter receptors (Figure S3); and calculation of the onset time of maximum intensity relative to full exposure to the compound (Figure S4) (PDF)

## Abbreviations Used

ATP: adenosine triphosphate
AUC: area under the curve
cAMP: cyclic adenosine monophosphate
CFP: cyan fluorescent protein
DCM: dual-channel microscope
EC50: half maximal effective concentration
FRET: Förster resonance energy transfer
fwhm: full width at half-maximum
GPCR: G-protein-coupled receptor
IP3: inositol triphosphate
PKA: protein kinase A
PLC: phospholipase C
TAS1R: taste receptor type 1
TAS2R: taste receptor type 2
YFP: yellow fluorescent protein
