# Supplementary material for: Tongue-on-a-Chip: Parallel Recording of Sweet and Bitter Receptor Responses to Sequential Injections of Pure and Mixed Sweeteners
Source: J Agric Food Chem. 2024 Jul 1;72(28):15854–64. doi: 10.1021/acs.jafc.4c00815 (PMC11261611; doi:10.1021/acs.jafc.4c00815)
Supplement: Supplementary file 1 — jf4c00815_si_001.pdf [file jf4c00815_si_001.pdf]

Supporting Information

Tongue-on-chip: Parallel recording of sweet and bitter receptor responses to sequential injections of pure and mixed sweeteners

Margriet Roelse*, Nadejda Krasteva, Steve Pawlizak, Michaela K. Mai, Maarten A. Jongsma

*T: +31 317 481084, email: margriet.roelse@wur.nl

Contents

Table S1, Effect of gene dose on the sweet taste receptor signalling using 2.5 mM aspartame as ligand, the values indicate the iRatio values after contrast with the blank 2

Table S2, Effect of gene dose on bitter taste receptor signalling, the values indicate the iRatio values after contrast with the blank..2

Figure S1, system calibration with Fluorescein dye 3

Figure S2, TAS1R2/R3 heterodimer gene coding plasmid dose 4

Figure S3, bitter receptor gene coding plasmid dose. 6

Figure S4, calculation of the onset time of maximum intensity relative to full exposure to the compound 7

Table S1, Effect of gene dose on the sweet taste receptor signalling using 2.5 mM aspartame as ligand, the values indicate the iRatio values after comparison to the blank

	**TAS2R3 in ng/μL**				
**TAS2R2 in ng/μL**	**16.7**	**8.3**	**4.2**	**2.1**	**1**
**16.7**	1.18	**1.20**	1.17	1.09	1.03
**8.3**	1.11	1.10	-	-	-
**4.2**	1.08	-	1.09	-	-
**2.1**	1.09	-	-	1.01	-
**1**	1.02	-	-	-	0.97

" – ", not determined

Table S2, Effect of gene dose on bitter taste receptor signalling; the values indicate the iRatio values after contrast with the blank

	**Gene dose range in ng/μL**						
**Receptor**	**66.7**	**33.3**	**16.7**	**8.3**	**4.2**	**2.1**	**Ligands and dose**
**TAS2R3**	-	1.09	**1.14**	1.01	1.03	-	**Chlorpheniramine 1000 μM**
**TAS2R8***	-<br>**1.29**	1.16<br>1.14	1.11<br>1.10	1.02<br>-	1.01<br>-	-<br>-	**Chloramphenicol 125 μM**
**TAS2R14***	-<br>**1.04**	**1.21**<br>1.03	1.15<br>1.02	1.11<br>-	1.06<br>-	-<br>-	**Picrotoxinin 15.6 μM**
**TAS2R31/R44/R53***	1.00<br>-	1.03<br>-	1.05<br>-	**1.09**<br>**1.05**	-<br>1.04	-<br>1.01	**Diphenidol 500 μM**
**TAS2R43/R52**	**1.12**	1.08	1.04	1.03	0.98	-	**Aristolochic acid 125 nM**
**TAS2R46 /R54**	**1.14**	1.08	1.06	1.08	1.02	-	**Strychnine 310 nM**

- Not determined

*Data from two separate experiments

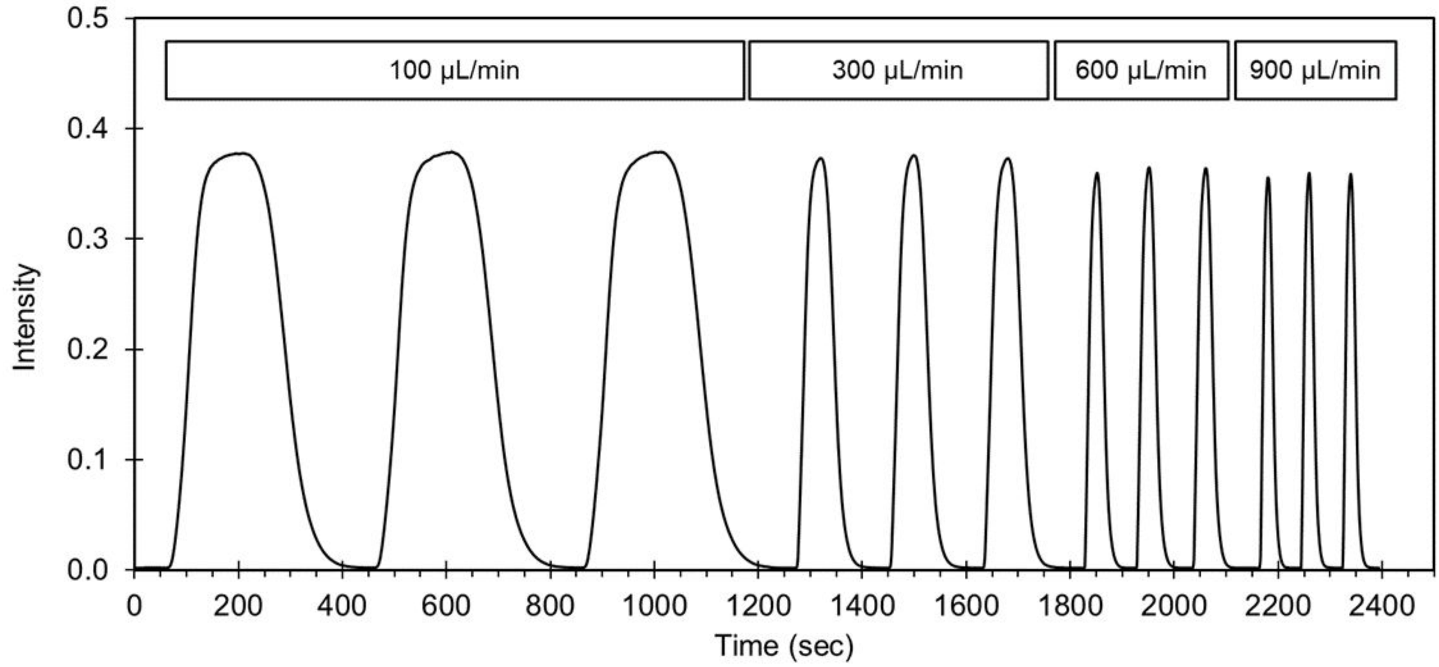

Figure S1, system calibration with Fluorescein dye. Triple injection of Fluorescein in an empty flowcell with internal volume of 100 μL and with a continuous flow of 100, 300, 600, and 900 μL/min using the pressure-controlled flow system of Figure 1. The variation in peak width (FWHM) is 1 s for 100 μL/min, 0.2 s for 600 μL/min, and there is no peak width variation for 300 and 900 μL/min. The variation in peak rise is between 0.2 and 0.9% and the variation in peak fall is between 0.01 and 0.7%.

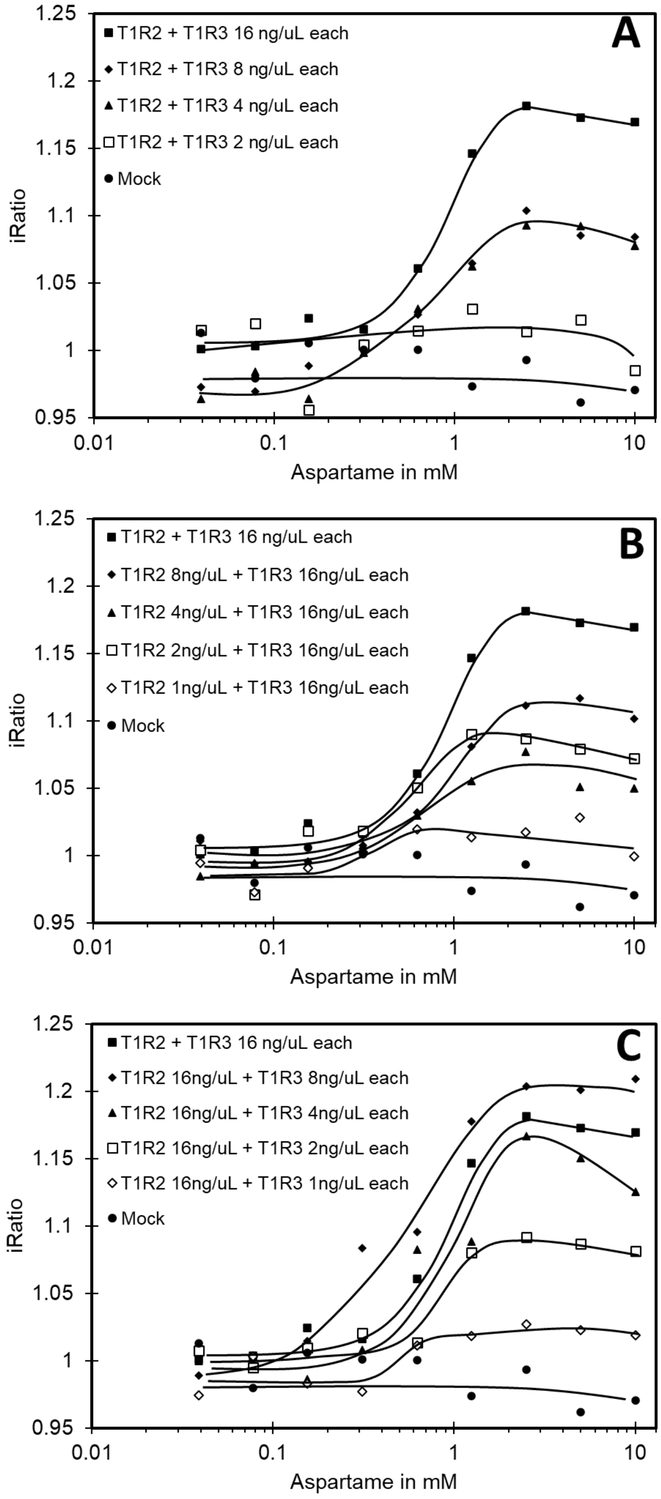

Figure S2, TAS1R2/R3 heterodimer gene coding plasmid dose. The optimal gene dose of the sweet receptor was determined in a reverse-transfected cell array via a dose-response series of Aspartame. The dose-response series were measured in real time on a single cell array. (A) Dose-response series to aspartame with equimolar concentrations TAS1R2 and TAS1R3. The ratio was titrated by reducing the dose of either TAS1R2 (B) or TAS1R3 (C).

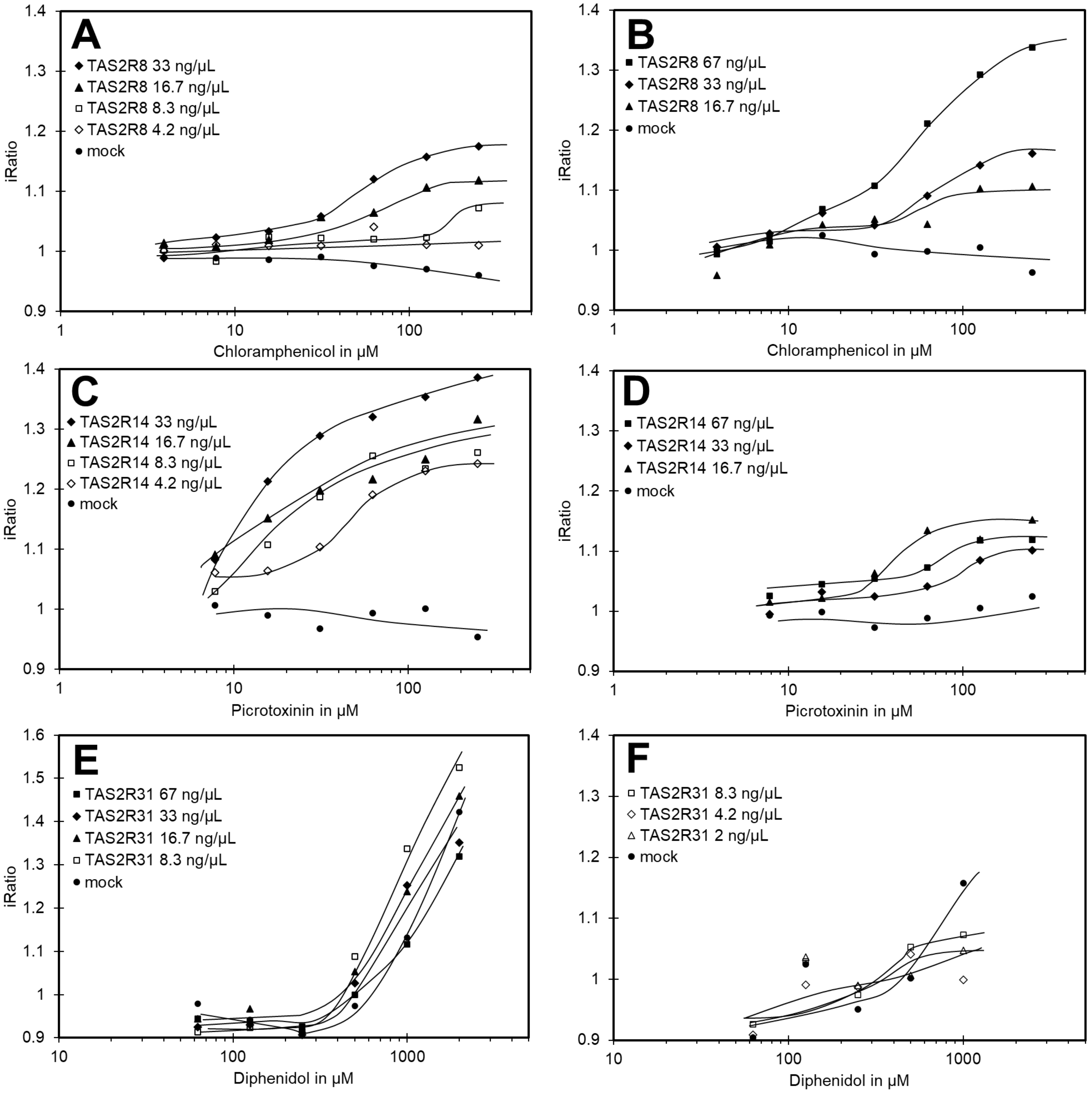
A
TAS2R8 33 ng/µL
TAS2R8 16.7 ng/µL
TAS2R8 8.3 ng/µL
TAS2R8 4.2 ng/µL
mock
iRatio
Chloramphenicol in µM
B
TAS2R8 67 ng/µL
TAS2R8 33 ng/µL
TAS2R8 16.7 ng/µL
mock
iRatio
Chloramphenicol in µM
C
TAS2R14 33 ng/µL
TAS2R14 16.7 ng/µL
TAS2R14 8.3 ng/µL
TAS2R14 4.2 ng/µL
mock
iRatio
Picrotoxinin in µM
D
TAS2R14 67 ng/µL
TAS2R14 33 ng/µL
TAS2R14 16.7 ng/µL
mock
iRatio
Picrotoxinin in µM
E
TAS2R31 67 ng/µL
TAS2R31 33 ng/µL
TAS2R31 16.7 ng/µL
TAS2R31 8.3 ng/µL
mock
iRatio
Diphenidol in µM
F
TAS2R31 8.3 ng/µL
TAS2R31 4.2 ng/µL
TAS2R31 2 ng/µL
mock
Diphenidol in µM

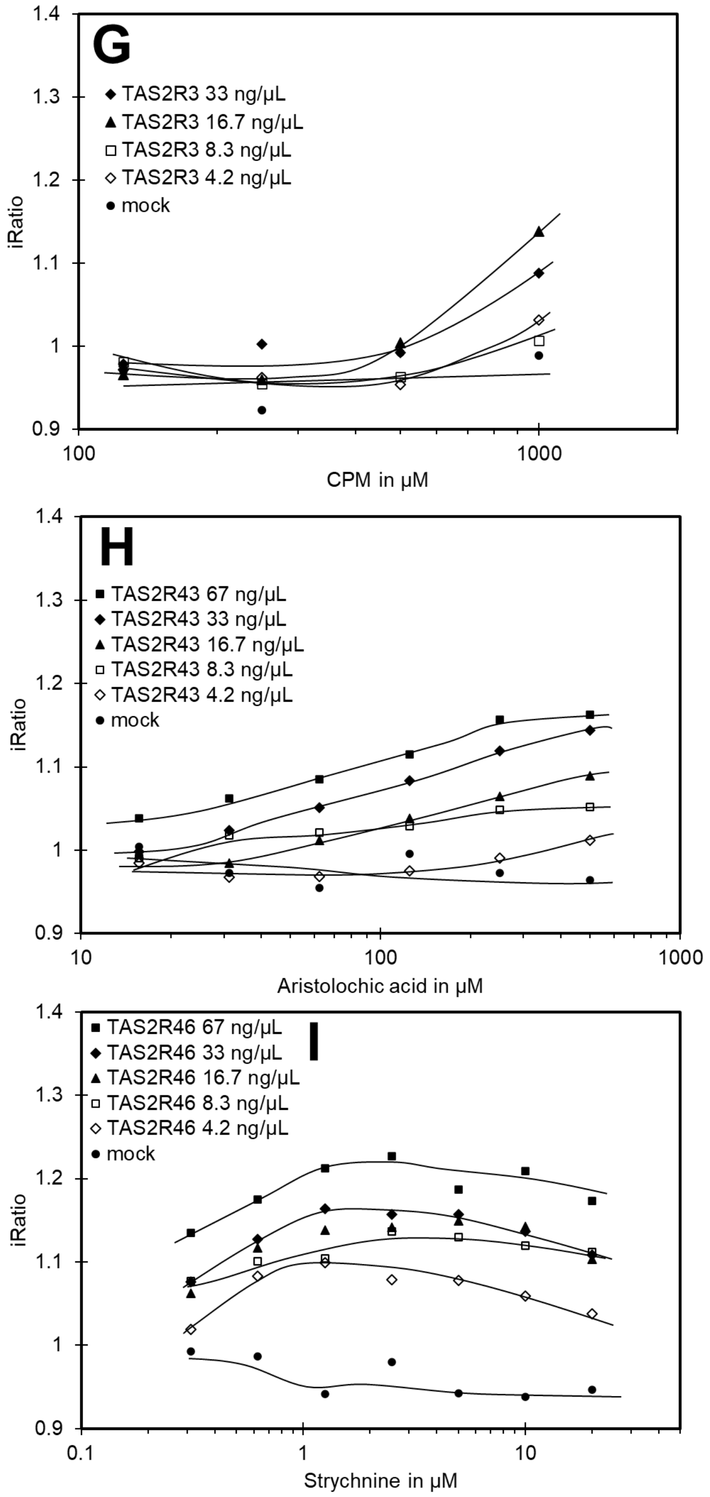

Figure S3, bitter receptor gene coding plasmid dose. The optimal gene dose of the bitter receptors was determined in a reverse transfected cell array via a dose-response series of each ligand. The dose-response series was measured in real time on a single chip per bitter receptor. (A,B) TAS2R8 dose-response with chloramphenicol, (C,D) TAS2R14 dose-response with picrotoxinin, (E,F)

TAS2R31 dose-response with diphenidol, (G) TAS2R3 dose-response with CPM (chloropheniramine), (H) TAS2R43 dose-response with aristolochic acid, and (I) TAS2R46 dose-response with strychnine.

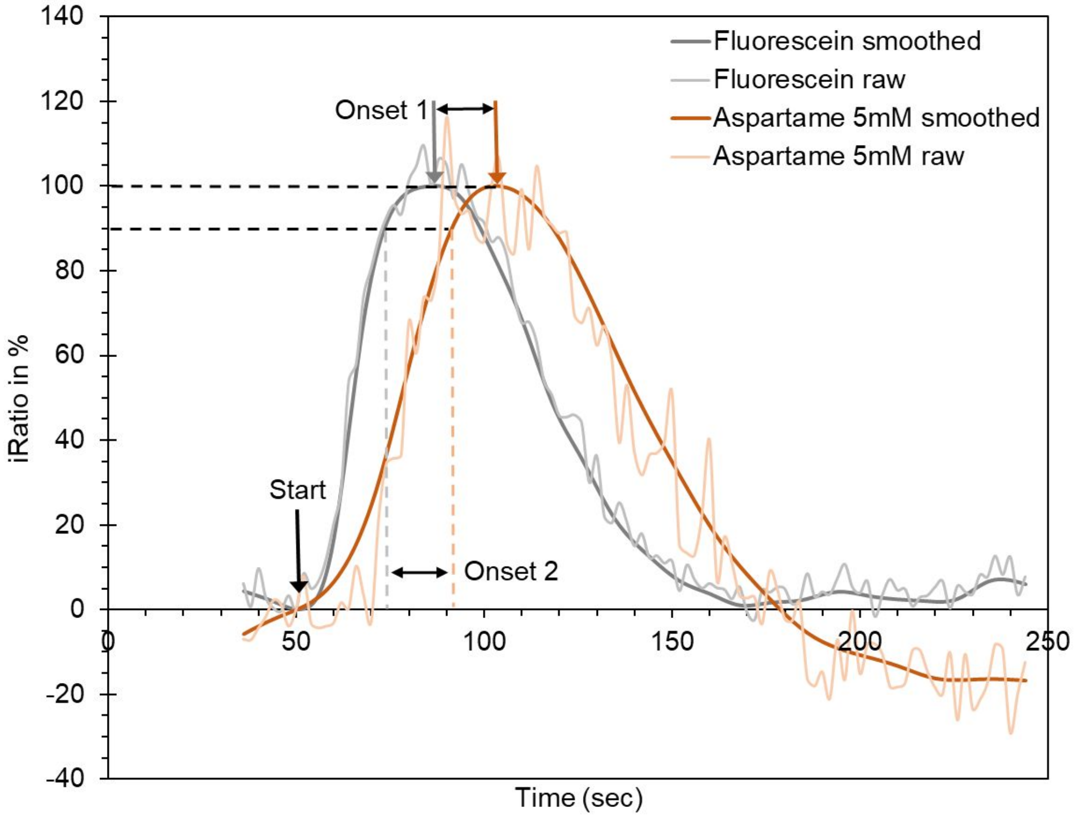

Figure S4, calculation of the onset time of maximum intensity relative to full exposure to the compound. There are two ways of calculating onset: Onset 1 equals the time between the concentration maximum of the ligand and the delay to reaching the response maximum. Onset 2 is based on the rise up to 90% of the maximum and might be considered if there is a considerable plateau broadening of the peaks and consequently the onset time, resulting in lower agreement to the experience of human panels.
